# The Association between Body Composition Phenotype and Insulin Resistance in Post-COVID-19 Syndrome Patients without Diabetes: A Cross-Sectional, Single-Center Study

**DOI:** 10.3390/nu16152468

**Published:** 2024-07-30

**Authors:** Dulce González-Islas, Laura Flores-Cisneros, Arturo Orea-Tejeda, Candace Keirns-Davis, Nadia Hernández-López, Laura Patricia Arcos-Pacheco, Andrea Zurita-Sandoval, Frida Albarran-López, Luis García-Castañeda, Fernanda Salgado-Fernández, Samantha Hernández-López, Angelia Jiménez-Valentín, Ilse Pérez-García

**Affiliations:** 1 Heart Failure and Respiratory Distress Clinic, Instituto Nacional de Enfermedades Respiratorias “Ismael Cosío Villegas”, Mexico City 14080, Mexico; gzz.dulce@gmail.com (D.G.-I.);; 2 Department of Epidemiological Information Analysis, General Directorate of Epidemiology, Mexico City 01480, Mexico; lauraflores.cisneros@gmail.com; 3 Licenciatura en Nutriología, Facultad de Estudios Superiores Zaragoza, Universidad Nacional Autónoma de México, Mexico City 09230, Mexico; 4 Clinical Nutrition Department, Hospital General “Dr. Manuel Gea González”, Mexico City 14080, Mexico

**Keywords:** body composition phenotype, insulin resistance, metabolic abnormalities, post-COVID-19, dynapenic obesity, obesity, sarcopenia

## Abstract

Background: The most frequent body composition alterations in post-COVID-19 syndrome include low muscle mass, dynapenia, sarcopenia, and obesity. These conditions share interconnected pathophysiological mechanisms that exacerbate each other. The relationship between body composition phenotypes and metabolic abnormalities in post-COVID-19 syndrome remains unclear. Objective: To evaluate the association between body composition phenotypes and insulin resistance (IR) and metabolic abnormalities in non-diabetic individuals with post-COVID-19 syndrome. Methods: A cross-sectional, single-center study involving 483 subjects with post-COVID-19 syndrome following moderate to severe acute COVID-19 requiring hospitalization. Individuals with diabetes, those who declined to participate, or those who could not be contacted were excluded. Body composition phenotypes were classified as normal weight, dynapenia, sarcopenia, dynapenic obesity, and sarcopenic obesity (SO). Results: The average age was 52.69 ± 14.75 years; of note, 67.08% were male. The prevalence of body composition phenotypes was as follows: 13.25% were of normal weight, 9.52% had dynapenia, 9.94% had sarcopenia, 43.69% had obesity, 18.84% had dynapenic obesity, and 4.76% had SO. Additionally, 58.18% had IR. Obesity (OR: 2.98, CI95%; 1.64–5.41) and dynapenic obesity (OR: 4.98, CI95%; 1.46–6.88) were associated with IR. Conclusion: The most common body composition phenotypes were obesity, dynapenic obesity, and dynapenia. Furthermore, obesity and dynapenic obesity were associated with IR in post-COVID-19 syndrome.

## 1. Introduction

Post-COVID-19 syndrome is characterized by symptoms and abnormalities that persist for more than 12 weeks and may continue for years after the initial acute viral infection caused by SARS-CoV-2 which cannot be attributed to other conditions [1]. The persistence of signs and symptoms is linked to the severity of the original COVID-19 infection [2]. The global prevalence of post-COVID-19 syndrome is 43%, with 54% of cases occurring in hospitalized subjects and 34% in non-hospitalized individuals [3]. The most frequently observed manifestations affect the pulmonary, hematologic, cardiovascular, endocrine, renal, gastrointestinal, and musculoskeletal systems [4].

The most characteristic musculoskeletal and body composition alterations in post-COVID-19 syndrome are fatigue, low muscle mass, dynapenia, sarcopenia, and obesity [2,5,6,7,8]. Dynapenia is defined as low muscle strength and can be assessed based on handgrip strength (HGS), which correlates with strength in other body compartments. Sarcopenia, on the other hand, is a progressive and generalized skeletal muscle disorder [9]. Additionally, body composition phenotypes such as low muscle mass, dynapenia, sarcopenia, and obesity increase the risk of adverse outcomes such as falls, physical disability, fractures, limited capacity for activities of daily living, impaired mobility, and mortality in diverse populations [10,11,12,13,14].

Several mechanisms may contribute to changes in body composition, including advanced age and pre-existing metabolic and pro-inflammatory conditions such as diabetes and pulmonary and cardiovascular diseases. During the acute phase of COVID-19, several factors contribute to changes in body composition, including the severity of the disease, anorexia, malnutrition, prolonged hospitalization, inflammatory states, oxidative stress, increased protein breakdown, the use of corticosteroids, and the use of mechanical ventilation and neuromuscular blockers in patients requiring intensive care, which can negatively impact respiratory muscles such as the intercostal muscles, diaphragm, and peripheral muscles [5,15,16,17].

Following SARS-CoV-2 infection, metabolic abnormalities have been observed, such as an increased risk of new-onset diabetes and hyperglycemia [18], as well as a higher prevalence of dynapenia, sarcopenia, and obesity [19,20,21].

Moreover, low muscle mass/strength, particularly sarcopenia, and obesity share strongly interconnected pathophysiological mechanisms that aggravate each other, creating a vicious cycle. These mechanisms include age-related reductions in protein synthesis and increased proteolysis, fat infiltration into skeletal muscle promoting lipotoxicity and aggravating inflammation, mitochondrial dysfunction, oxidative stress [22,23,24], and metabolic abnormalities such as insulin resistance (IR), which is commonly defined as diminished sensitivity or receptivity to the metabolic actions of insulin and is a risk factor for metabolic syndrome (MetS) and type 2 diabetes [25,26].

Excess adipose tissue and low muscle mass/strength coexist in sarcopenic obesity (SO), which has been associated with adverse outcomes [27]. Furthermore, previous studies have shown a relationship between body composition alterations and metabolic abnormalities, particularly IR, in diverse populations [28,29].

Despite the prevalence of post-COVID-19 syndrome and the observed metabolic abnormalities following SARS-CoV-2 infection, the relationship between body composition phenotypes and metabolic abnormalities in post-COVID-19 syndrome has not been fully examined. Therefore, this study aimed to evaluate the association between body composition phenotypes and IR, as well as metabolic abnormalities in subjects with post-COVID-19 syndrome without diabetes.

## 2. Materials and Methods

### 2.1. Study Design and Population

A cross-sectional single-center study was conducted at the Instituto Nacional de Enfermedades Respiratorias “Ismael Cosío Villegas” in Mexico City. Data were obtained from outpatient evaluations conducted and clinical records examined three months after SARS-CoV-2 infection as part of routine clinical examinations of post-COVID-19 subjects. The study period spanned from 1 June 2020 to 30 May 2023.

**Figure 1:**
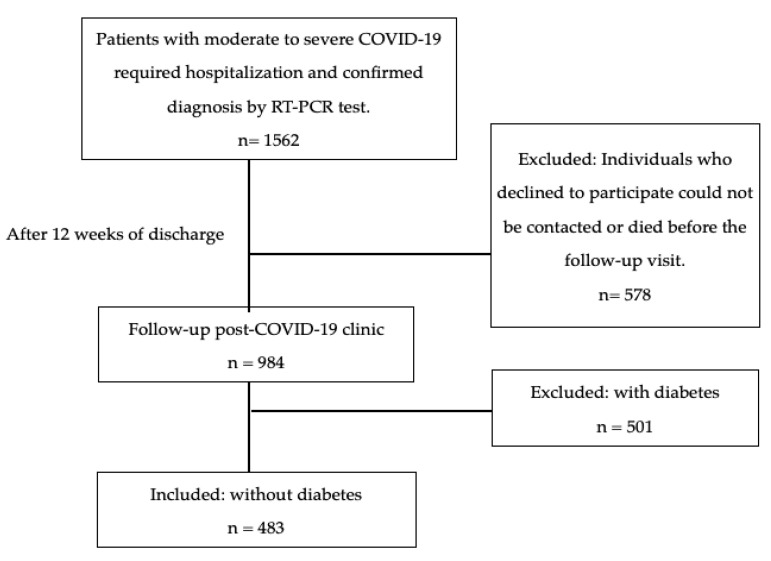
Study flowchart diagram.

Patients were invited to participate in the follow-up post-COVID clinic around 12 weeks after hospital discharge. Subjects who required hospitalization during the acute phase due to moderate to severe COVID-19, with a confirmed diagnosis via PCR testing, were included. Individuals with diabetes, those who declined to participate, or those who could not be contacted were excluded from the study (Figure 1).

#### Outcome Measures

Clinical information and data about hospitalization for SARS-CoV-2 were obtained from medical records. Body composition, anthropometry, metabolic parameters, and clinical and demographic variables were measured as part of the post-COVID-19 clinical management implemented for the patients who came to our hospital.

### 2.2. Insulin Resistance (IR)

IR was defined as a Homeostatic Model Assessment of Insulin Resistance Index (HOMA-IR) > 2.5 [30].

### 2.3. Metabolic Syndrome (MetS)

MetS was assessed using the modified National Cholesterol Education Program Adult Treatment Panel (NCEP ATP III) criteria. MetS was defined as the presence of three or more of the following points: (1) elevated blood pressure (systolic blood pressure > 130 mmHg or diastolic blood pressure > 85 mmHg), (2) hypertriglyceridemia (triglycerides > 150 mg/dL), (3) low HDL cholesterol (HDL cholesterol < 50 mg/dL in men, <40 mg/dL in women), (4) hyperglycemia (plasma glucose > 100 mg/dL or treatment for diabetes), and (5) obesity (in men, fat mass > 25% and ≤16 kg; in women, fat mass > 35%) [31].

### 2.4. Anthropometry

Weight and height measurements were performed in accordance with the manual anthropometric standardization reference [32]. All of the subjects wore light clothing and were barefoot. Body mass index (BMI) was calculated by dividing weight (kilograms) by height squared (meters).

### 2.5. Handgrip Strength (HGS)

According to Rodríguez-García et al. [33], HGS was evaluated using a mechanical Smedley Hand Dynamometer (Stoelting, Wood Dale, UK).

### 2.6. Body Composition

Body composition was measured with whole-body bioelectrical impedance using four-pole mono-frequency equipment, the RJL Quantum IV analyzer (RJL Systems^®^, Clinton Township, MI, USA). Using a technique previously described [34], fat mass was estimated using RJL Systems’ software BC 4.2.2.

Appendicular skeletal muscle mass adjusted by Height^2^ (ASM/Height^2^) was assessed according to Sergi’s formula [35]: ASM/Height^2^ (Kg/m^2^) = [−3.964 + (0.227 * (Height^2^ (cm)/Resistance) + (0.095 * Weight) + (1.384 * Sex) + (0.064 * Reactance)/Height (m^2^)].

### 2.7. Body Composition Phenotype

Normal weight was defined as adequate muscle mass (in men, ASM/Height^2^ > 7.0 kg/m^2^ and in women, ASM/Height^2^ > 6.0 kg/m^2^), muscle strength (in men, HGS > 27 kg in men and in women, HGS > 16 kg), and fat mass (≤25% in men and ≤35% in women) [9,36].

Dynapenia was defined as low muscle strength conforming to the European Working Group on Sarcopenia in Older People 2 (EWGSOP2) [9], with thresholds of ≤27 kg HGS in men and ≤16 kg HGS in women.

Sarcopenia was defined according to EWGSOP2 [9] as low muscle mass (in men, ASM/Height^2^ ≤ 7.0 kg/m^2^ and in women, ASM/Height^2^ ≤ 6.0 kg/m^2^) and low muscle strength (in men, HGS ≤ 27 kg and in women, HGS ≤ 16 kg).

Obesity was defined as fat mass > 25% in men and > 35% in women [36]. Dynapenic obesity was defined in men as low muscle strength (HGS ≤ 27 kg in men and women) and excess adiposity (fat mass > 25% in men and >35% in women) [9,36].

Sarcopenic obesity (SO) was defined as low muscle mass (in men ASM/Height^2^ ≤ 7.0 kg/m^2^ and ≤6.0 kg/m^2^ in women), low muscle strength (HGS ≤ 27 kg and ≤16 kg in women), and excess adiposity (in men fat mass > 25% and in women as fat mass > 35%) [9,36].

### 2.8. Statistical Analysis

Analyses were conducted using the commercially available STATA package, version 14 (Stata Corp., College Station, TX, USA). Categorical variables were expressed as frequencies and percentages. Normality in continuous variables was evaluated using the Shapiro–Wilk test. Normal continuous variables were expressed as the mean and standard deviation, while non-normal variables were expressed as the median and interquartile range (25th–75th percentiles). Comparisons among the study groups were analyzed using the Chi-square test for categorical variables and t-tests or Mann–Whitney U tests for continuous variables.

A simple logistic regression model was performed to evaluate the risk factors associated with insulin resistance (IR), and the odds ratios (OR) and 95% confidence intervals (CI) were estimated. The multivariate logistic regression model was adjusted for variables that exhibited significance at *p* < 0.20 in the crude model. Potential interactions and multicollinearity between variables were tested. A *p*-value < 0.05 was considered statistically significant.

## 3. Results

A total of 483 subjects diagnosed with post-COVID-19 syndrome were assessed, with a mean age of 52.69 ± 14.75 years; of note, 67.08% were male. Among the subjects, 13.25% were of normal weight, 9.52% had dynapenia, 9.94% had sarcopenia, 43.69% had obesity, 18.84% had dynapenic obesity, and 4.76% had SO. The insulin-resistant group (HOMA-IR > 2.5) was younger, with higher weight, height, BMI, HGS, phase angle, fat mass, and obesity and a lower prevalence of dynapenia and sarcopenia compared to the non-insulin-resistant group (Table 1).

### 3.1. Metabolic Alterations

**Figure 2:**
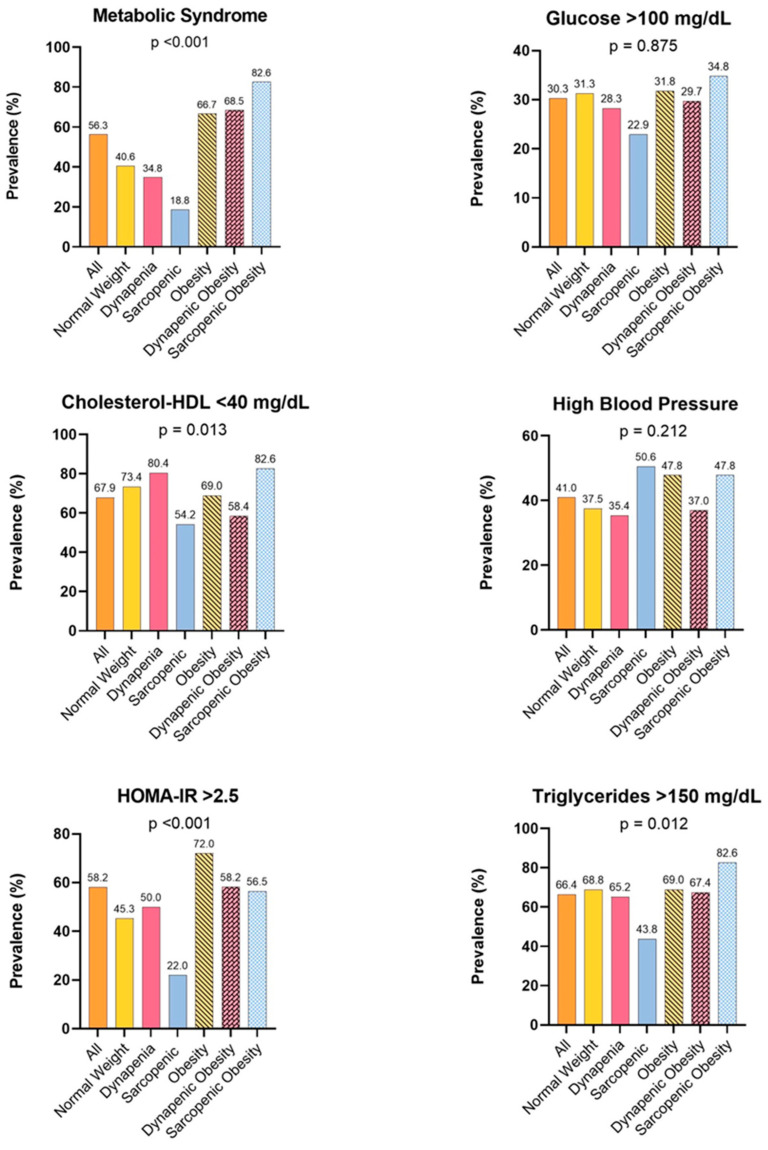
Prevalence of metabolic alterations according to body composition phenotype.

Regarding metabolic alterations, 56.33% had MetS, 58.18% had IR (HOMA-IR > 2.5), 30.23% had glucose > 100 mg/dL, 67.86% had HDL cholesterol < 40 mg/dL, 40.99% had high blood pressure, and 66.38% had triglycerides > 150 mg/dL. Figure 2 shows the metabolic alterations among the different body composition phenotypes.

### 3.2. Risk Factors Associated with IR

Regarding risk factors associated with IR, a bivariate model showed that age (OR: 0.97, CI 95%: 0.96 to 0.98, *p* < 0.001), COPD (OR: 0.22, CI 95%: 0.07 to 0.71, *p* = 0.012), length of hospital stay (OR: 0.98, CI 95%: 0.97 to 0.99, *p* = 0.001), weight (OR: 1.04, CI 95%: 1.02 to 1.05, *p* < 0.001), height (OR: 1.02, CI 95%: 1.00 to 1.04, *p* = 0.013), BMI (OR: 1.12, CI 95%: 1.08 to 1.16, *p* < 0.001), HGS (OR: 1.03, CI 95%: 1.01 to 1.05, *p* = 0.001), phase angle (OR: 1.32, CI 95%: 1.13 to 1.52, *p* < 0.001), ASM/Height^2^ (OR: 1.37, CI 95%: 1.17 to 1.60, *p* < 0.001), fat mass % (OR: 1.05, CI 95%: 1.03 to 1.07, *p* < 0.001), obesity (OR: 3.10, CI 95%: 1.74 to 5.53, *p* < 0.001), and dynapenic obesity (OR: 3.75, CI 95%: 1.15 to 12.21, *p* = 0.028) are associated with IR (Table 2).

**Figure 3:**
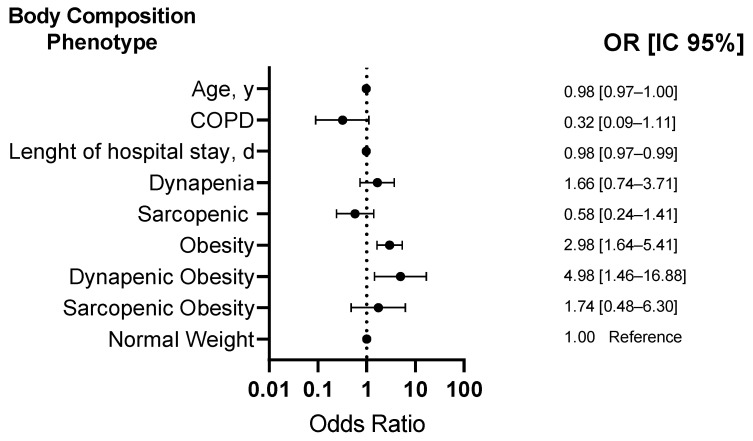
Association between body composition phenotype and insulin resistance.

Regarding body composition phenotypes associated with IR, a multivariate model showed that subjects with obesity (OR: 2.98, CI 95%: 1.64 to 5.41, *p* < 0.001) and dynapenic obesity (OR: 4.98, CI 95%: 1.46 to 16.88, *p* = 0.01) are associated with a higher risk of IR after adjusting for age, COPD, and hospital stay (Figure 3).

## 4. Discussion

One of the main findings of our research highlights the association between body composition phenotypes and the risk of IR, as well as the prevalence and metabolic profiles across these body composition phenotypes in individuals with post-COVID-19 syndrome.

In our study population, a high prevalence of alterations in body composition and metabolic parameters was observed, with only 13.25% of individuals classified as having a normal weight.

Concerning muscle strength, 9.52% had dynapenia and 18.84% had dynapenic obesity. Dynapenia refers to a low level of muscle strength and functionality. It has been associated with lower pulmonary function [37], prolonged hospital stays [38], and mortality [12] in various populations. Moreover, Wilkinson and Cols. found that individuals with reduced muscle strength had a 1.63 times higher risk of hospitalization or death from COVID-19 [39]. Within the context of post-COVID-19 syndrome, there is a high prevalence of dynapenia. López-Sampalo et al. reported that nearly 80% of patients over 65 years age exhibited dynapenia three months after infection, with a prevalence of 57% persisting at 12 months [7]. Another study observed a prevalence of dynapenia of 17.5% in outpatient individuals [8].

Another body composition phenotype commonly observed in individuals with post-COVID-19 syndrome is sarcopenia, characterized by low muscle mass and strength. In patients with post-COVID-19 syndrome, the prevalence of sarcopenia can vary depending on population characteristics and the diagnostic method used. For example, LoMauro et al. found a 41% prevalence of sarcopenia, as assessed using the SARC-F questionnaire, in individuals with neurological and motor deficits [21]. In contrast, a study by Lopez-Sampalo et al. reported a prevalence of 32% at three months and 19% at twelve months of follow-up among both hospitalized and ambulatory older adults, also using the SARC-F questionnaire. Our study found a prevalence of sarcopenia of 9.94% and 4.76% of SO, according to EWGSOP2 [9].

In severe or critical COVID-19 survivors, a prevalence of obesity higher than 60% has been observed [40]. Additionally, in subjects with post-COVID-19 syndrome who did not require hospitalization, the prevalence of obesity appears to be lower at 28% [8]. However, the coexistence of obesity with dynapenia or sarcopenia was not evaluated in these populations. One contribution of our study is that we assessed the coexistence of obesity with dynapenia or sarcopenia, demonstrating a negative impact on the quality of life and prognosis of the subjects [39].

SARS-CoV-2 infection contributes to body composition alterations, including loss of muscle strength and mass and myosteatosis. These changes may be attributed to factors such as increased oxidative stress, inflammatory cytokines, and the use of glucocorticoids. These factors could promote higher accumulation of adipose tissue and more severe manifestations [17,22,23,24,41,42,43,44], as IR and MetS [10] increase the risk of cardiovascular events and all-cause mortality. Additionally, low skeletal muscle mass and intramuscular fat accumulation contribute to impaired muscle contractility [28,45].

Several studies have shown that patients who have recovered from COVID-19 exhibit alterations in body composition and metabolism [7,8]. Another contribution of our study was to evaluate the association between fat mass, muscle/strength mass, and IR using a multivariate analysis, which allows for adjustment for confounding variables. Our study revealed that obese individuals had a 2.98-fold higher risk of IR. However, this risk significantly increased in individuals with dynapenic obesity (OR: 4.98, 95% CI: 1.46–16.88), while SO (OR: 1.74, 95% CI: 0.48–6.30) did not show statistical significance, likely due to the smaller sample size.

Skeletal muscle is an organ implicated in insulin-induced glucose metabolism mediated by glucose transporter 4 (GLUT4), which accounts for approximately 80% of glucose clearance [46]. A decline in muscle mass has been related to IR, MetS, and diabetes [47,48]

In the context of IR, compensatory hyperinsulinemia occurs, leading to reduced suppression of glycogenesis, accelerated protein catabolism, decreased protein anabolism, and increased myostatin levels [49,50]. IR combined with muscle loss inhibits β-oxidation, promotes increased lipolysis, and releases free fatty acids, leading to the aggregation of triglycerides in skeletal muscle and the liver [49,51]. Moreover, adipose tissue produces pro-inflammatory cytokines such as TNF-α, IL-6, and C-reactive protein, which negatively impact IR and are linked with lower muscle mass and diminished muscle strength [52,53]. Our results show that subjects with IR had shorter hospital stays; this may be because these subjects were younger, which is also reflected in higher strength and ASM/height^2^.

Concerning MetS and IR, higher prevalences were found in patients with obesity, dynapenic obesity, and OS. Although the prevalence of metabolic disorders in the population was high, 56.33% had MetS and 58.18% had IR.

Although the number of SARS-CoV-2 infections has decreased considerably, the sequelae in these subjects continue, especially in those with moderate to severe disease. The findings of this study highlight the need to screen both body composition and metabolic variables in patients who present with COVID-19 to apply therapeutic measures to mitigate and reverse body composition and metabolic alterations, preventing adverse events and improving quality of life.

### Strengths and Limitations

This study has inherent limitations due to its cross-sectional design, which makes it impossible to assess causality and the incidence of different body compositions. Nonetheless, it also exhibits notable strengths. The diagnosis of body composition phenotypes was based on electrical bioimpedance analysis, which allows for estimating different body compartments such as muscle and fat mass. Furthermore, this study considers the coexistence of dynapenia and sarcopenia with obesity, which carries significant implications.

## 5. Conclusions

In post-COVID-19 subjects, there is a high prevalence of metabolic alterations and changes in body composition, such as obesity and dynapenic obesity. Additionally, both obesity and dynapenic obesity were found to be associated with a higher risk of IR.

## Figures and Tables

**Table 1:** Demographic and clinical characteristics according to insulin resistance in post-COVID-19 syndrome patients without diabetic subjects.

	All *n* = 483	HOMA-IR > 2.5 *n* = 281	HOMA-IR < 2.5 *n* = 202	*p*-Value
Age, y	52.69 ± 14.75	50.4 ± 14.04	55.89 ± 15.16	<0.001
Male, *n* (%)	324 (67.08)	188 (66.90)	136 (67.33)	0.922
**Co-morbidities**				
Hypertension, *n* (%)	153 (31.68)	90 (32.03)	63 (31.19)	0.845
Ischemic cardiopathy, *n* (%)	32 (6.63)	17 (6.05)	15 (7.43)	0.549
Pulmonary disease, *n* (%)	75 (15.53)	39 (13.88)	36 (17.08)	0.238
Thyroid disease, *n* (%)	32 (6.63)	20 (7.12)	12 (5.94)	0.608
Hepatopathy, *n* (%)	8 (1.66)	3 (1.07)	5 (2.48)	0.232
HIV, *n* (%)	7 (1.45)	4 (1.42)	3 (1.49)	0.955
Asthma, *n* (%)	13 (2.69)	8 (2.85)	5 (2.48)	0.803
COPD, *n* (%)	16 (3.31)	4 (1.42)	12 (5.94)	0.006
**Hospitalary parameters**				
Hospital stay, d	17 [10,11,12,13,14,15,16,17,18,19,20,21,22,23,24,25,26,27,28,29]	15 [6,7,8,9,10,11,12,13,14,15,16,17,18,19,20,21,22,23,24,25,26]	20 [6,7,8,9,10,11,12,13,14,15,16,17,18,19,20,21,22,23,24,25,26,27,28,29,30,31,32,33,34]	0.006
Mechanical ventilation, *n* (%)	289 (60.33)	163 (58.63)	126 (62.69)	0.371
**Body composition**				
Weight, kg	81.6 ± 18.8	86.6 ± 17.98	74.60 ± 17.80	<0.001
Height, cm	163.47 ± 9.63	164.40 ± 8.78	162.18 ± 10.59	0.012
BMI, kg/m^2^	30.44 ± 6.37	32.01 ± 6.05	28.27 ± 6.17	<0.001
Handgrip strength, kg	24.85 ± 9.87	26.11 ± 9.41	23.10 ± 10.24	<0.001
Prediction marker, 200/5 kHz	0.79 ± 0.05	0.79 ± 0.05	0.79 ± 0.05	0.811
Phase angle, °	6.01 ± 1.52	6.24 ± 1.41	5.71 ± 1.60	<0.001
ASM/height^2^, kg/m^2^	7.79 ± 1.40	8.01 ± 1.12	7.48 ± 1.67	<0.001
Fat mass, kg	30.08 ± 10.70	32.23 ± 8.84	27.09 ± 12.26	<0.001
**Body composition phenotype**				
Normal weight, *n* (%)	64 (13.25)	29 (10.32)	35 (17.33)	<0.001
Dynapenia, *n* (%)	46 (9.52)	23 (8.19)	23 (11.39)	
Sarcopenia, *n* (%)	48 (9.94)	11 (3.91)	37 (18.32)	
Obesity, *n* (%)	211 (43.69)	152 (54.09)	59 (29.21)	
Dynapenic obesity, *n* (%)	91 (18.84)	53 (18.86)	38 (18.81)	
Sarcopenic obesity, *n* (%)	23 (4.76)	13 (4.63)	10 (4.95)	

HIV—human immunodeficiency virus; COPD—chronic obstructive pulmonary disease; BMI—body mass index; ASM/height^2^—appendicular skeletal muscle mass adjusted by height.

**Table 2:** Risk factors associated with insulin resistance (HOMA-IR > 2.5) in post-COVID-19 syndrome patients without diabetes. Crude model.

	OR	95% CI	*p*-Value
Age, y	0.97	0.96 to 0.98	<0.001
Male	0.98	0.66 to 1.44	0.922
**Co-morbidities**			
Hypertension	1.03	0.70 to 1.53	0.845
Ischemic cardiopathy	0.80	0.39 to 1.64	0.549
Pulmonary disease	0.74	0.45 to 1.21	0.239
Thyroid disease	1.21	0.57 to 2.54	0.608
Hepatopathy	0.42	0.10 to 1.79	0.245
HIV	0.95	0.21 to 4.32	0.955
Asthma	1.15	0.37 to 3.58	0.804
COPD	0.22	0.07 to 0.71	0.012
**Hospitalary parameters**			
Length of hospital stay, d	0.98	0.97 to 0.99	0.001
Mechanical ventilation	0.84	0.58 to 1.22	0.371
**Body composition**			
Weight, kg	1.04	1.02 to 1.05	<0.001
Height, cm	1.02	1.00 to 1.04	0.013
BMI, kg/m^2^	1.12	1.08 to 1.16	<0.001
Handgrip strength, kg	1.03	1.01 to 1.05	0.001
Prediction marker, 200/5 kHz	0.55	0.004 to 69.75	0.810
Phase angle, °	1.32	1.13 to 1.52	<0.001
ASM/height^2^, kg/m^2^	1.37	1.17 to 1.60	<0.001
Fat mass, %	1.05	1.03 to 1.07	<0.001
**Body composition phenotype**			
Normal weight	1	Reference	
Dynapenia	1.20	0.56 to 2.57	0.627
Sarcopenia	0.35	0.15 to 0.82	0.016
Obesity	3.10	1.74 to 5.53	<0.001
Dynapenic obesity	3.75	1.15 to 12.21	0.028
Sarcopenic obesity	1.11	0.32 to 3.81	0.862

HIV—human immunodeficiency virus; COPD—chronic obstructive pulmonary disease; BMI—body mass index; ASM/heigth^2^—appendicular muscular mass adjusted by height.

## Data Availability

The data presented in this study are available on request from the corresponding author due ethical reasons.
